# Case Report: Dual molecular diagnosis of gain-of-function *STAT1* mutation and regulatory *STAT3* variant in a patient with a hyper-IgE-like phenotype

**DOI:** 10.3389/fimmu.2025.1646761

**Published:** 2025-10-03

**Authors:** Roukaya Yaakoubi, Najla Mekki, Amel Ben Chehida, Ansem Benhammadi, Koon-Wing Chan, Daniel Leung, Charfeddine Gharsallah, Fatma Zahra Guerfali, Mohamed-Ridha Barbouche, Yu Lung Lau, Meriem Ben-Ali, Imen Ben-Mustapha

**Affiliations:** ^1^ Laboratory of Transmission, Control and Immunobiology of Infections, Institut Pasteur de Tunis, University Tunis El-Manar, Tunis, Tunisia; ^2^ Department of medicine, University of Rochester Medical Center, Rochester, NY, United States; ^3^ Faculty of Medicine of Tunis, Tunis El-Manar University, Tunis, Tunisia; ^4^ Department of Pediatrics, La Rabta Hospital, Tunis, Tunisia; ^5^ Department of Pediatrics and Adolescent Medicine, Li Ka Shing Faculty of Medicine, The University of Hong Kong, Hong Kong, Hong Kong SAR, China; ^6^ Institut Pasteur, Université Paris Cité, INSERM U1201, Unité de Parasitologie Moléculaire et Signalisation, Paris, France; ^7^ Department of Microbiology, Immunology and Infectious Diseases, College of Medicine and Medical Sciences, Arabian Gulf University, Manama, Bahrain

**Keywords:** STA3-GOF, STAT1-GOF, dual molecular diagnosis, hyper IgE syndrome, CMC

## Abstract

**Background:**

The transcription factors signal transducer and activator of transcription 1 and 3 (STAT1 and STAT3) play essential roles in immune and non-immune cell function. The clinical characterization of patients carrying germline gain or loss-of-function (GOF or LOF) mutations in these genes has significantly improved our understanding of their physiological and pathological roles. Although patients with *STAT3* LOF, *STAT3* GOF, and *STAT1* GOF mutations are classified into distinct inborn errors of immunity (IEI) categories, namely Hyper-IgE Syndrome, Regulatory T cell defects, and predisposition to Mucocutaneous Candidiasis, respectively, there is notable clinical overlap among these disorders.

**Case summary:**

We describe a 17-year-old girl with recurrent lung infection leading to bronchiectasis, chronic onychomycosis, recurrent vulvovaginal candidiasis, and oral thrush. Additional findings included short stature, delayed puberty, and retained primary teeth. Laboratory results revealed eosinophilia and elevated IgE serum levels, with a NIH HIES score of 53. A rare heterozygous deletion within the 3′UTR of the *STAT3* gene (c.*351_*353del) was identified through a candidate gene approach. Although the variant is in a non-coding region, increased *STAT3* phosphorylation and elevated suppressor of cytokine signaling 3 (SOCS3) expressions suggested a potential GOF effect. *In silico* analysis further predicted that the deletion disrupts microRNA (miRNA) binding sites and RNA binding proteins (RBP), potentially impairing post-transcriptional regulation and contributing to *STAT3* overexpression. Given the complexity of the phenotype and the atypical location of the *STAT3* variation, whole-exome sequencing (WES) was performed, revealing a heterozygous missense mutation in the *STAT1* DNA-binding domain (*c.1053G>T*, *p.L351F*), previously reported in autosomal dominant chronic mucocutaneous candidiasis (AD-CMC). Functional assays on lymphocytes confirmed an increased *STAT1* phosphorylation compared to both *STAT1* LOF patient and healthy controls.

**Conclusion:**

This case highlights the diagnostic complexity of overlapping IEI phenotypes and the value of combining targeted and WES strategies. This dual molecular diagnosis, comprising a regulatory variant in *STAT3* and a pathogenic coding mutation in *STAT1*, emphasizes the need to include non-coding regions in genetic analyses. It also underscores the value of using techniques that offer a broader genomic view and capture all coding exons, enabling a more comprehensive correlation with the clinical and immunological phenotype.

## Introduction

The Hyper IgE Syndromes (HIES) are a rare group of inborn errors of immunity (IEI) characterized by eczema, recurrent skin and lung infections, and elevated serum IgE levels (1, 2). Advances in genetic testing allowed the identification of various monogenic disorders underlying HIES. Dominant negative (DN) loss-of-function (LOF) mutations in *STAT3* gene represent the first identified and most prevalent genetic cause of HIES worldwide, commonly referred to as Job’s syndrome (OMIM 147060). Patients with autosomal dominant (AD) mutations in *STAT3* (AD-STAT3) typically develop eczematoid dermatitis, recurrent pneumonias, bacterial skin infections, and chronic mucocutaneous candidiasis (CMC). Non-immunological defects, including connective tissue and skeletal abnormalities are also prominent. Retained primary teeth have been documented in around 70% of AD-STAT3 individuals (3). Laboratory findings of AD-STAT3 include elevated IgE serum levels, eosinophilia, and decreased TH17 cells. The diagnosis of HIES due to *STAT3* mutations is typically guided by the NIH-HIES scoring system, with a score above 30 (4). Ultimately, final confirmation of AD-STAT3 relies on genetic sequencing, which remains the gold standard for identifying causative mutations. While heterozygous LOF mutations in the *STAT3* gene have been well documented as the cause of Job’s syndrome, it was not until 2014 that heterozygous gain-of-function (GOF) mutations in the same gene were identified as causing a distinct immune dysregulation disorder primarily characterized by autoimmunity and autoinflammation (5, 6).AD-STAT3 GOF mutations can present with recurrent infections, asthma, along with growth delay in half of patients. Dental abnormalities, a common feature of AD-STAT3, were noted in 4.2% of cases (7). STAT3-LOF and STAT3-GOF share several key features with other IEI, including atopic symptoms, and susceptibility to fungal and bacterial infections, similar to what is observed in AD-STAT1 GOF disorders. This latter is primarily associated with severe CMC mainly resulting from impaired TH17 cell function (8, 9).

We report herein, a Tunisian patient with a clinical phenotype consistent with the diagnosis of HIES, primarily characterized by recurrent pulmonary and fungal infections and harboring a dual molecular diagnosis involving a GOF regulatory variant in *STAT3* and a GOF mutation in *STAT1*.

## Case description

The patient is a Tunisian girl, born to consanguineous parents following an uneventful pregnancy. Her family history was unremarkable, with the exception of a paternal uncle reported to have asthma and infertility. She was referred to the pediatric department at the age of 7 for the evaluation of recurrent lung infections which had been occurring almost fortnightly since she was two months old. These infections were associated with severe poorly controlled asthma and relapsing severe dermatitis of the face and the diaper area. Symptoms persisted despite the control of environmental triggers, high doses of inhaled beclomethasone (up to 1000 µg/day) and salbutamol as needed. Gastro-esophageal reflux (GERD) was diagnosed by ultrasonography and treated with domperidone, although pH-metry was normal. Allergic skin tests conducted after discontinuation of corticosteroids were negative. The patient also suffered from chronic relapsing oral candidiasis, which was variably treated with oral amphotericin, nystatin or miconazole. At the age of 7 years, the time of the referral, she presented with chronic cough and bronchial hypersecretion. Growth parameters were within the normal limits. Physical examination revealed diffuse wheezing, digital clubbing without chest deformities and severe eczema involving the face and the trunk. Additionally, she had severe and chronic fingers onychomycosis characterized by paronychia, nail thickening, yellowish discoloration, and onycholysis. These findings were associated with oral, vulvar and vaginal candidiasis. Chest radiography showed diffuse cystic and cylindric bronchiectases. Cardiac echography and weat chloride concentration were normal (18,8 mmol/l; NV < 60 mmol/l for the later).

Complete blood count revealed eosinophilia (1160 cells/mm^3^). Immunoglobulin levels and lymphocyte phenotyping were within the normal ranges at that time. Lymphocyte proliferation to phytohemagglutinin (PHA) was normal, but markedly reduced in response to specific antigens (**Table 1**). Human immunodeficiency virus serology was negative. Serum gamma globulin and alpha1 globulin levels were normal (12,9g/l; NV = 8-13,5 g/l and 3,83g/l; NV = 2,1-3,5 g/l respectively). Spirometry showed a distal obstruction reversible after salbutamol inhalation. The patient was initially managed with inhaled salbutamol, high doses of inhaled corticosteroids, chest physiotherapy and prophylactic sequential antibiotics (erythromycin, amoxicillin, cotrimoxazole-trimethoprim). Topical econazole nitrate and oral nystatin were prescribed. Due to frequent asthma exacerbations, occurring fortnightly, her treatment was adjusted at the age of 13 years to fluticasone propionate (500µg/day) and salmeterol (50µ/day). Symptoms of allergic rhinitis were treated with beclomethasone (400 µg/day) and Cetirizine (10 mg/day). Asthma and seasonal rhinitis were partially controlled due to poor compliance to inhaled treatment. At the age of 14, she had chronic mycosis of fingernails treated with systemic fluconazole, recurrent upper respiratory infections, pneumonias and multiple furuncles, all managed with oral antibiotics. Short stature and weight loss had been noticed since the age of ten years. Her growth curve and bone age were not in favor of a growth hormone deficit. Coeliac disease, renal failure, chronic anemia, tubulopathy and hypothyroidism were excluded. There was no evidence of autoimmunity, and thyroid autoantibodies were negative. Serum IgE immunoglobulin levels were elevated (>1000 U/ml). After optimizing asthma control and ensuring compliance with a hypercaloric diet, a trend toward pubertal catch-up growth was observed between the ages of 15 and 17. No fractures, skeletal abnormalities, or hyperextensibility were documented, however, high palate and retention of two primary teeth requiring dental extractions were noted.

**Table 1:** Immune profile of the patient during follow-up.

	7 years	18 years	20 years
Lymphocytes (10^3^cells/µl)	2.39 (3.90-9)	**-**	2.506 (1400-3300)
*Lymphocytes subsets (normal range)*			
CD3+ (%) (cells/µl)	83.5 (51-77) -	71 (56-84) -	98.74 (56-84) 1909 (1000-2200)
CD3+ CD4+ (%) (cells/µl)	57 (35 - 56) -	40.9 (31-52) -	58.88 (31/52) 1.124 (530-1300)
CD3+ CD8+ (%) (cells/µl)	28 (12 - 23) -	47 (18-35) -	35.04(18-35) 669 (330-920)
CD19+ (%) (cells/µl)	12 (11 - 41) -	**0.5 (6-23) ↘** **-**	**0.08 (6-23) ↘** **2 (110-570) ↘**
CD16+ CD56+ (%) (cells/µl)	4.5 (0.3-14) -	**1.5 (3-22) ↘** **-**	**1 (3-22) ↘** **20 (70-480) ↘**
T *CD4 cell subsets* (%)			
TEMRA CD3+/CD4+/CCR7-/CD45RA+)	–	**-**	0.2 (0.0083-6.8)
NAIVE (CD3+/CD4+/CCR7+/CD45RA+)	–	**-**	16 (16-100)
TEM (CD3+/CD4+/CCR7-/CD45RA-)	–	**-**	**25 (1-23)**
TCM (CD3+/CD4+/CCR7+/CD45RA-)	–	**-**	58.8 (18-95)
CXCR5+ memory helper T cells §	–	**-**	13.4 (5-56)
T CD8 cell subsets (%)			
TEMRA (CD3+/CD8+/CCR7-/CD45RA+)	–	**-**	11.5 (7-53)
NAIVE (CD3+/CD8+/CCR7+/CD45RA+)	–	**-**	54.3 (6-100)
TEM (CD3+/CD8+/CCR7-/CD45RA-)	–	**-**	30.3 (14-98)
TCM (CD3+/CD8+/CCR7+/CD45RA-)	–	**-**	3.9 (1-20)
T CD4+/CD25+/CD127-	–	**-**	**3.3 (4-14)↘**
*Immunoglobulin levels g/L (normal range)*			
IgG	**16.84 (6.46-14.51)**	**-**	13.07 (6.58-18.37)
IgA	1.74 (0.57-2.04)	**-**	2.64 (0. 17-3.60)
IgM	0.88 (0.44-2.42)	**-**	0.5 (0.40-2.63)
IgG1	**-**	7.92 (3.824-9.286)	**-**
IgG2	**-**	**2.12 (2.418-7.003) ↘**	**-**
IgG3	**-**	1.02 (0.2182-1.7606	**-**
IgG4	**-**	0.082 (0.0392-0.864)	**-**
*Proliferation tests (cpm/IS)*			
PHA	237380/79	**-**	**-**
Anti-CD3	40661/10.5	**-**	**-**
Tetanus	**5665/0.5 ↘**		
PPD	**10841/2↘**	**-**	**-**
Antinuclear antibodies	**-**	**-**	**Negative**

Bold values are abnormal.

## Diagnostic assessment

At the age of 13, the patient was referred to the national reference laboratory of IEI at Pasteur Institute of Tunis, in Tunisia, for expanded investigation. Based on her clinical history, HIES was suspected. A NIH HIES score of 53 further supported the diagnosis of AD-STAT3 deficiency (Supplemental file).

**Figure 1:**
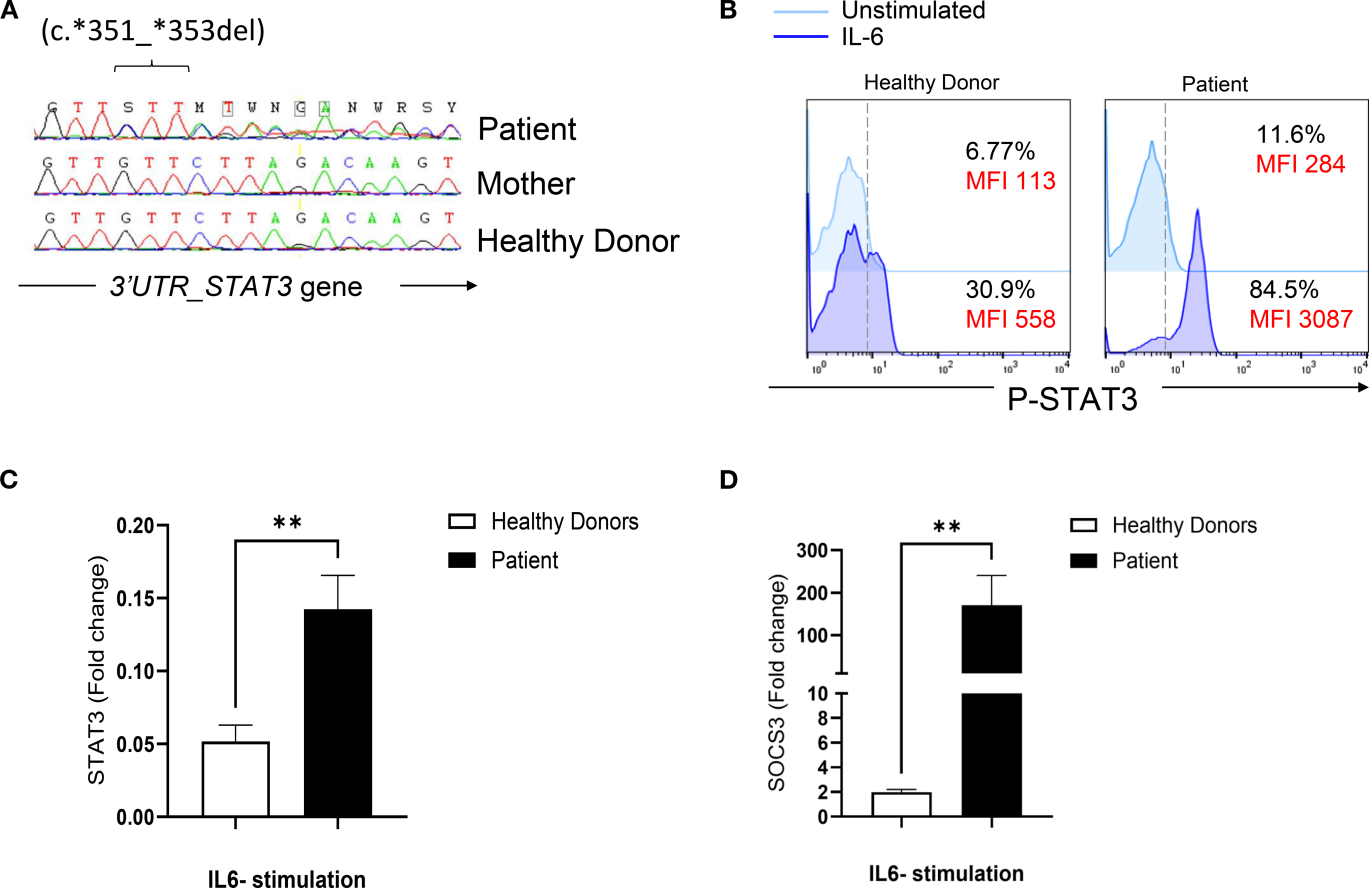
Genetic and functional evaluation of a STAT3 variant in a patient with a suspected HIES phenotype. **(A)** Sanger sequencing chromatogram showing a heterozygous deletion (c.351_353del) in the 3’ untranslated region (UTR) of STAT3. The variant is present in the patient and absent (wild-type) in the mother and a healthy control. **(B)** Flow cytometric analysis of STAT3 phosphorylation in PBMCs stimulated with IL-6 (20 ng/mL) for 20 mn in the patient and a healthy donor. **(C)** Quantitative RT-PCR analysis of STAT3 mRNA levels in PBMCs stimulated with IL-6 (100 ng/mL) for 2 hours in the patient and healthy donors. **(D)** Expression of SOCS3, a downstream target of STAT3, measured by quantitative RT-PCR in PBMCs stimulated with IL-6 (100 ng/mL) for 2 hours in the patient and healthy donors. Statistical significance was assessed using an unpaired t-test.

Sanger sequencing of *STAT3* gene (ENST00000264657.10) did not reveal any mutations in the coding exons. However, a rare heterozygous variant located in the 3′ untranslated region (c.*351_*353del) was identified (rs533596827) (**Figure 1A**). This deletion was absent in 100 alleles from 50 healthy Tunisian donors (data not shown).

To evaluate the functional impact of this variant, STAT3 phosphorylation was assessed in the patient’s PBMCs and found to be elevated in response to Interleukin (IL)-6 stimulation compared to healthy donors (**Figure 1B**). In addition, transcript levels of STAT3 and its downstream target, suppressor of cytokine signaling 3 (SOCS3), were upregulated after IL-6 stimulation (**Figures 1C, D**). These findings suggest that the variant likely has a GOF effect.

We performed a bioinformatic analysis to investigate whether the 3′UTR variant might disrupt microRNA (miRNA)-mediated regulation of *STAT3*. Using miRDB (MicroRNA Target Prediction Database) and TargetScanHuman 7.2, we identified two miRNAs, hsa-miR-3529-3p and hsa-miR-196a-1-3p, with predicted binding sites within the *STAT3* 3′UTR that are likely abolished as a result of the deletion. Moreover, to further assess the impact of the GTT motif deletion in the 3′UTR of STAT3, we analyzed the regulatory elements within this UG-rich sequence (AUUGUUGUUGUU-351GUU353-CUUAGA) affected by the deletion. As shown in **Supplementary Figure S1**, bioinformatic predictions using RBP map, POSTAR3, and ENCODE datasets indicate that this mutation specifically disrupts the binding of several RNA binding proteins (RBPs), while others RBP that bind outside this region are likely unaffected.

**Figure 2:**
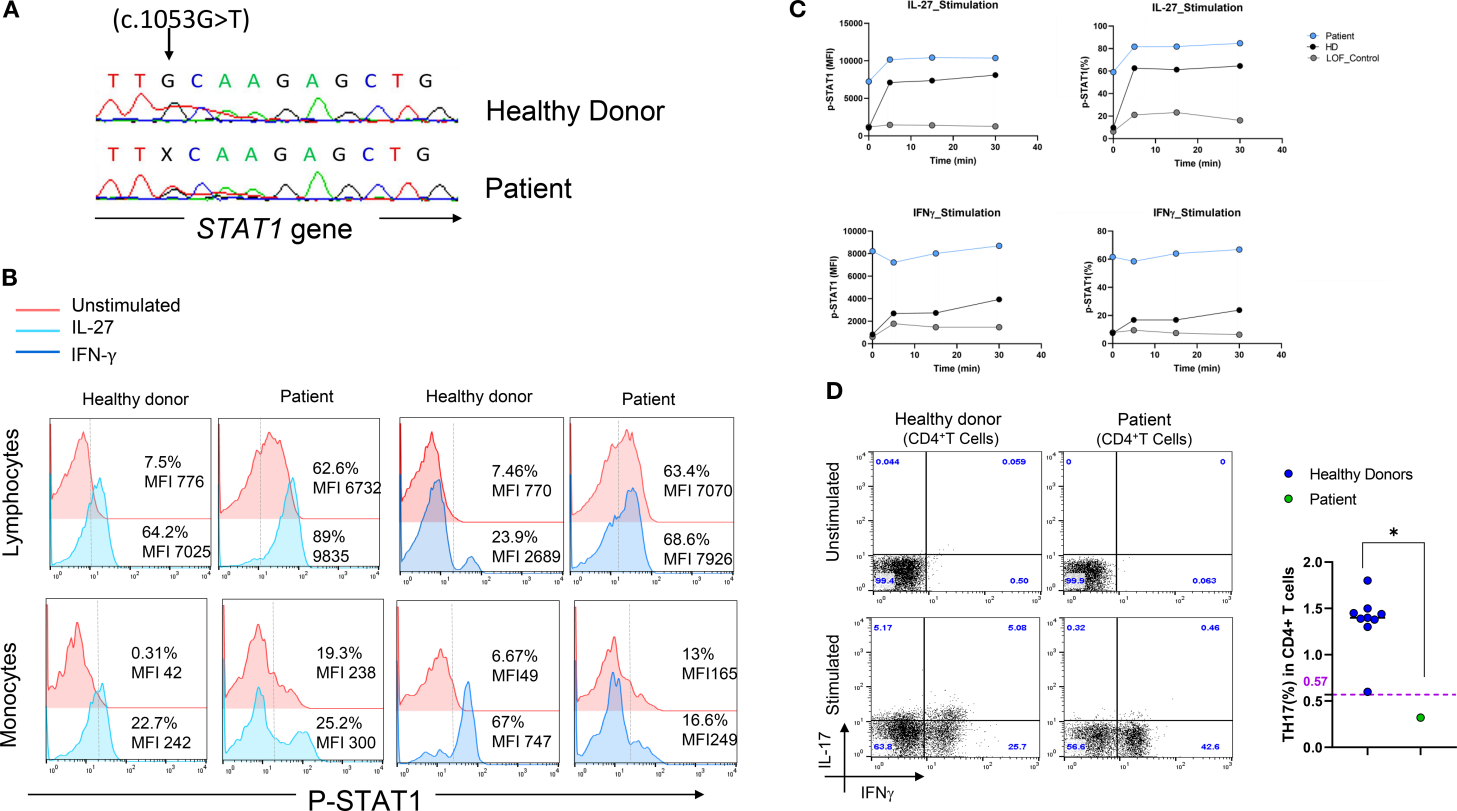
Genetic and functional characterization of a STAT1 mutation in a patient with a suspected HIES phenotype. **(A)** Sanger sequencing chromatogram confirming the heterozygous *STAT1* mutation (c.1053G>T, p.L351F) identified by WES. **(B)** STAT1 phosphorylated (pSTAT1) in the patient’s lymphocytes and monocytes at baseline and after stimulation with either IL-27 (100 ng/mL, left panel) or IFN-γ (100 ng/mL, right panel) for 15 minutes, compared to a healthy control. **(C)** STAT1 phosphorylation kinetics following stimulation with IL-27 (left panel) or IFN-γ (right panel), showing enhanced phosphorylation in the patient’s lymphocytes (gain-of-function, GOF) compared to a *STAT1* loss-of-function (LOF) patient and a healthy donor. **(D)** Ex vivo evaluation of IL-17-producing T cells (CD4^+^ IL-17^+^ IFN-γ⁻) in nonadherent PBMCs as determined by flow cytometry: Non-adherent cells were stimulated for 12 hours with PMA and ionomycin in the presence of Golgi Plug. After cell-surface staining of the CD4+ T cells, cells were fixed, permeabilized and stained with anti-IL-17A antibody. (left panel). Percentage of TH17 cells in the patient compared to healthy controls. Horizontal bars represent median values (right panel).

Given the uncommon location of the variant and the complexity of the patient’s clinical and immunological phenotype, whole-exome sequencing (WES) was performed at the age of 17, revealing a previously reported mutation in the DNA-binding domain of *STAT1* (c.1053G>T, p. L351F) (**Figure 2A**) (10, 11). Flow cytometric analysis of STAT1 phosphorylation (pSTAT1) revealed elevated levels of pSTAT1 in the patient’s lymphocytes compared to controls, both at baseline and following IL-27 or IFN-γ stimulation (**Figure 2B**). Further assessment of pSTAT1 kinetics showed that patient’s lymphocytes exhibited sustained and higher phosphorylation levels in response to IL-27 and IFN-γ over time, in contrast to cells from a STAT1-LOF patient or control cells (**Figure 2C**). Assessment of pSTAT1 in monocytes revealed a distinct pattern: IL-27–induced pSTAT1 stimulation in the patient’s monocytes was slightly increased compared to HD, whereas the amplitude of IFN-γ–induced pSTAT1 in CD14^+^ monocytes was lower than in HD (**Figure 2B**).

Extended immunophenotyping of PBMCs was performed and revealed a reduced percentage of TH17 cells compared to healthy controls (**Figure 2D**). During follow-up at the age of 20, immunophenotyping revealed significant B cell lymphopenia despite normal immunoglobulin levels, along with a reduced Natural Killer (NK) cells count (**Table 1**).

## Discussion

We report herein, a dual molecular diagnosis of GOF variants in both *STAT3* and *STAT1* in a patient with a HIES-like phenotype. The elevated NIH score of 53 including non-immunologic manifestations shaped a clinical picture reminiscent of a STAT3 deficiency. Candidate gene strategy identified a heterozygous deletion (c.*351_*353del) in the 3’UTR region of *STAT3* gene. Increased STAT3 phosphorylation along with increased transcript levels of *SOCS3* gene both at baseline and after IL-6 stimulation suggested a GOF effect. This rare variant with a MAF < 0.01 is located within a regulatory region and may lead to altered post-transcriptional regulation of STAT3. Such dysregulation could disrupt the tight physiological control normally exerted by regulatory proteins such as protein inhibitors of activated STATs (PIAS) and SOCSs, which prevent excessive STAT3 activation (12). Recent studies have shown that long non-coding RNAs and microRNAs can directly inhibit STAT3 activity through sequence complementarity with STAT3 at the 3′-UTR, 5′-UTR, or coding regions (13, 14). In this context, the variant identified in our patient was hypothesized to interfere with a microRNA-mediated post-transcriptional regulation of *STAT3*. Bioinformatic analysis revealed that two miRNAs (hsa-miR-3529-3p and hsa-miR-196a-1-3p) are predicted to lose binding sites within the *STAT3* 3′UTR due to the deletion. Specifically, hsa-miR-3529-3p normally binds to three sites (positions 340, 443, and 352), with the site at position 352 containing four consecutive ‘GTT’ motifs critical for miRNA recognition. The detected deletion removes this motif, thereby abolishing the site and reducing the number of functional miRNA binding sites, which may impair post-transcriptional repression and lead to increased *STAT3* expression. A comparable disruption was observed for hsa-miR-196a-1-3p, which loses one of its four predicted binding sites at position 347. These *in silico* findings suggest that the 3′UTR deletion may attenuate miRNA-dependent negative regulation of *STAT3*, potentially explaining the elevated expression observed in our patient. Moreover, bioinformatic analysis showed that this mutation specifically disrupts the binding of several RNA binding proteins (RBPs) further supporting the hypothesis that the deletion within the STAT3 3′UTR functionally impacts its post-transcriptional regulation.

An unbiased WES approach was employed in light of the patient’s atypical clinical presentation which included abnormal fungal susceptibility but lacked the hallmark features typically associated with STAT3 GOF syndrome, such as lymphoproliferation, autoimmune cytopenia, and multisystem autoimmunity. This analysis identified a heterozygous mutation (c.1053G>T, p.L351F) in the DNA-binding domain (DBD) of the *STAT1* gene. This variant has previously been functionally characterized as a GOF mutation in individuals with CMC (10, 11). Accordingly, the patient showed increased and sustained levels of phosphorylated STAT1 in lymphocytes as compared to control. The GOF nature of the variant is also supported by the markedly elevated steady-state STAT1 phosphorylation, with approximately 60% of CD4^+^ T cells positive for pSTAT1 in the absence of stimulation, substantially higher than the <10% typically observed in healthy controls or in patients with STAT1 loss-of-function mutation. Constitutive STAT1 hyperphosphorylation is a well-established feature of STAT1 GOF mutations and has been associated with sustained phosphorylation kinetics and impaired Th17 differentiation (15, 16). The lack of further pSTAT1 increase upon IFN-γ stimulation observed in our patient likely reflects chronic *in vivo* STAT1 activation that triggers negative feedback regulation, receptor internalization, or pathway desensitization ex vivo, mechanisms previously reported in other STAT1 GOF cases (17). Indeed, persistent activation may reduce the dynamic range of STAT1 responsiveness by saturating signaling capacity at baseline, thereby dampening the relative fold induction upon cytokine challenge. These findings highlight the importance of assessing both basal and stimulated phosphorylation states when evaluating JAK–STAT pathway dysregulation in suspected GOF variants Interestingly, pSTAT1 assessment in monocytes showed a distinct pattern with slightly increased IL-27 induced but reduced IFN-γ–induced responses in the patient’s monocytes compared to HD. Thus, T cells retain hyper-responsiveness to both IL-27 and IFN-γ, whereas monocytes display selective attenuation of IFN-γ signaling. This could underlie a cell and/or pathway-specific alteration that could potentially involve the STAT3-GOF variant present in the same patient.

Immunophenotyping revealed marked B cell lymphopenia despite normal immunoglobulin level, an observation that may reflect the presence of a limited number of B cells restricted to lymphoid tissues, with few or no B cells detectable in peripheral blood (18). The patient also exhibited a reduced number of NK cells. This finding is consistent with previous reports of decreased NK cell counts in STAT1 GOF patients, which may contribute to their increased susceptibility to viral infections (8). However, no viral infections were documented in this patient. In light of the decreased NK cell count, regular monitoring will be implemented to promptly detect and manage any potential viral infections.

The patient also showed reduced percentages of TH17 cells. Indeed, STAT1 gain-of-function mutations are known to compromise Th17 cell–mediated immunity primarily by inhibiting the differentiation of naïve CD4^+^ T cells into Th17 cells (8). This effect results from hyperactive STAT1 signaling, which antagonizes STAT3-dependent pathway required for Th17 lineage commitment, rather than from an intrinsic functional defect in mature Th17 cells (19–21). Consequently, patients with STAT1 GOF mutations typically exhibit reduced frequencies of Th17 cells and diminished IL-17 production, reflecting a polarization defect. (8). IL-17 plays a critical role in antifungal defense at epithelial barriers. Consistently, our patient exhibited increased susceptibility to fungal infections, including severe and chronic onychomycosis, along with recurrent vulvar and vaginal yeast infections. Indeed, in the most extensive multicenter study to date, comprising 274 individuals from 167 families, CMC emerged as the predominant feature, affecting 98% of cases (15). While individuals with impaired IL-17 responses may also exhibit increased vulnerability to bacterial infections (22, 23). The patient described in this report experienced recurrent bacterial infections that led to bronchiectasis. Moreover, she presented a GERD which is not a primary feature of STAT1-GOF, but has been reported in other clinical cases (24). Furthermore, even though over one-third of patients with STAT1-GOF mutations have been reported to exhibit autoimmune manifestations, including those harboring the L351F variant, our patient showed no clinical or biological signs of autoimmunity (15, 25). STAT1 and STAT3 are members of the STAT family of transcription factors, both of which play pivotal roles in regulating immune responses. Although structurally related, they often exert opposing effects on cellular processes. STAT1 is primarily associated with pro-inflammatory signaling and the induction of apoptosis, whereas STAT3 promotes anti-inflammatory responses and cell survival. This functional opposition creates a yin–yang dynamic, in which the balance between STAT1 and STAT3 activities critically influences cell fate decisions such as survival versus apoptosis, or pro versus anti-inflammatory responses (21). Moreover, STAT1 and STAT3 can directly modulate each other’s activity through various mechanisms. For example, STAT1 can inhibit STAT3’s DNA binding and transcriptional activity, thereby dampening STAT3-driven survival signals. Conversely, STAT3 can suppress STAT1 activity by downregulating components of the interferon-stimulated gene factor 3 (ISGF3) complex (26). In this context, the gain-of-function (GOF) mutation in STAT3 observed in our patient may counterbalance the hyperactivation of the STAT1 pathway, potentially explaining the absence of autoimmune manifestations.

In addition, the presence of non-immunological features reminiscent of STAT3 LOF such as retained primary teeth, has only been reported once in a case of STAT1-GOF syndrome without further investigation into the potential link between STAT1-GOF mutations and dental anomalies (24).

Taken together, the ‘mixed’ clinical phenotype, characterized by abnormal fungal susceptibility, recurrent lower respiratory tract infections, eczema, GERD, short stature, pubertal delay and retained primary teeth does not represent a simple additive combination of the two classical presentations, but rather a composite profile shaped by a dynamic interplay between STAT1 and STAT3 pathways. This highlights the need to move beyond single-gene paradigms and to consider network-level interactions, particularly in the context of dual molecular diagnoses.

The patient was managed with antifungal prophylaxis to control CMC, inhaled corticosteroids for asthma, domperidone for gastroesophageal reflux, and antibiotic therapy for recurrent bacterial infections. Recently, Janus kinase (JAK) inhibitors have emerged as a promising treatment option, particularly in STAT1-GOF patients with prominent autoimmune manifestations or severe, refractory infections (27, 28). However, initiation of JAK inhibitor therapy was not considered in our case, given the absence of autoimmune features and the relatively manageable infections.

In summary, this report highlights the diagnostic challenges in a patient presenting with a hyper-IgE-like syndrome ultimately found to carry a dual molecular defects. The combined use of a candidate gene approach and WES proved essential, each method offering complementary insights. While WES enabled the identification of a pathogenic *STAT1* variant that accounted for key clinical features, the candidate gene strategy allowed the detection of a non-coding 3′UTR variant in *STAT3* that would likely have been missed by exome sequencing alone. In silico analyses suggest that this deletion may disrupt miRNA-mediated repression of *STAT3*, potentially contributing to STAT3 overexpression in the patient. These findings underscore the importance of integrating non-coding regions into the genetic evaluation of IEI, especially in complex phenotypes that cannot be fully explained by coding variants identified through standard whole-exome sequencing (WES) if proposed alone as a first-line approach.

## Data Availability

The original contributions presented in this study are included in the article and/or **Supplementary Material**. Further inquiries can be directed to the corresponding author.
