# Supplementary figures and images for: Case Report: Dual molecular diagnosis of gain-of-function STAT1 mutation and regulatory STAT3 variant in a patient with a hyper-IgE-like phenotype
Source: Front Immunol. 2025 Oct 3;16:1646761. doi: 10.3389/fimmu.2025.1646761 (PMC12531231; doi:10.3389/fimmu.2025.1646761)

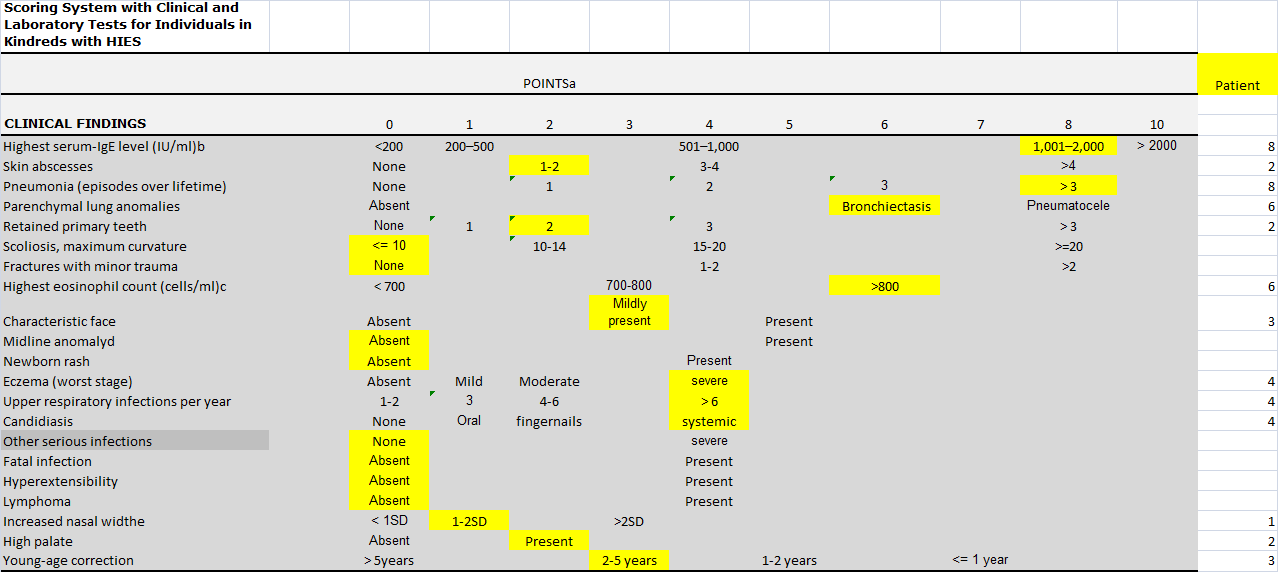


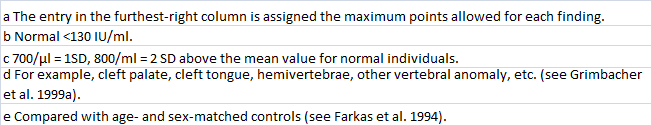

Supplement: Supplementary file 1 [file Table1.docx]
