# Supplementary material for: Case Report: Dual molecular diagnosis of gain-of-function STAT1 mutation and regulatory STAT3 variant in a patient with a hyper-IgE-like phenotype
Source: Front Immunol. 2025 Oct 3;16:1646761. doi: 10.3389/fimmu.2025.1646761 (PMC12531231; doi:10.3389/fimmu.2025.1646761)
Supplement: Supplementary file 2 [file Supplementaryfile1.docx]

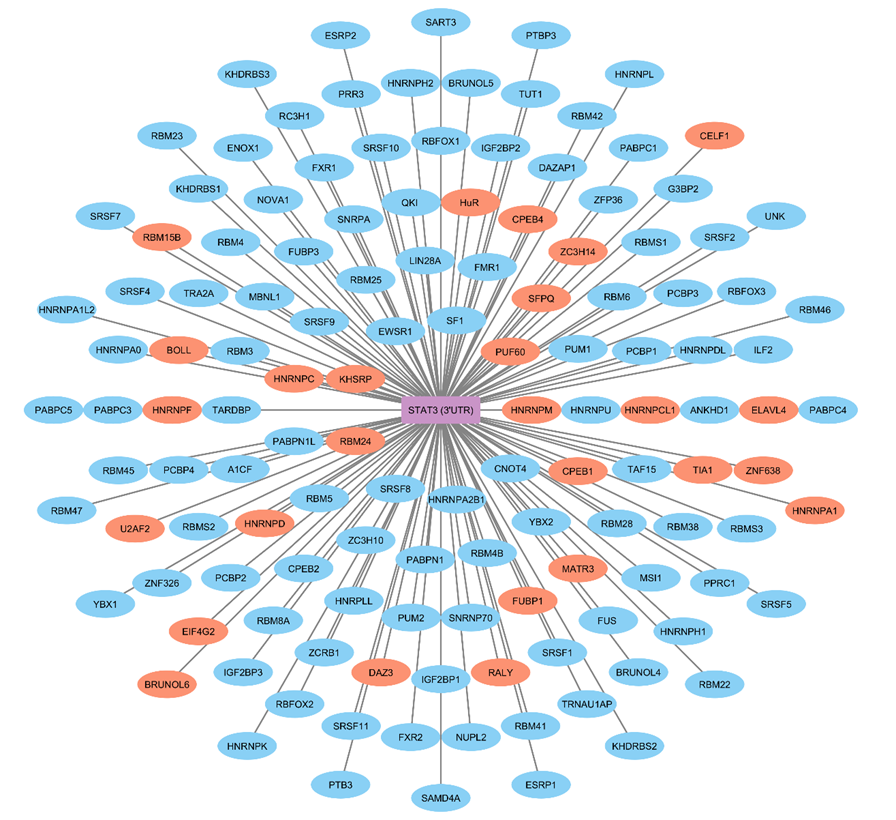


**Figure S1: Predictive analysis of RNA-binding protein (RBP) interactions with the STAT3 3'UTR region via RBPmap, POSTAR3, and ENCODE.**

Red-labeled proteins: RBPs with binding sites that directly overlap the UG-rich sequence (AUUGUUGUUGUU-351GUU353-CUUAGA) affected by the GTT motif deletion; their interaction with STAT3 is likely disrupted by this mutation. Blue-labeled proteins: RBPs that bind to regions of STAT3 mRNA outside the deletion site; their interaction is likely unaffected by the GTT mutation.
